# Supplementary material for: A ‘hidden problem’: Nature, prevalence and factors associated with sexual dysfunction in persons living with HIV/AIDS in Uganda
Source: PLoS One. 2024 Mar 7;19(3):e0295224. doi: 10.1371/journal.pone.0295224 (PMC10919729; doi:10.1371/journal.pone.0295224)
Supplement: Attachment — Submitted filename: Peer review comments on PONE-D-22-24725_.pdf [file pone.0295224.s001.pdf]

General

Abstract, sentence starting with 'Among individuals living with HIV/AIDS…': Suggest adding changing to '…have hardly been studied, especially in sub-Saharan Africa…'

[Lack of] hyphenation is a recurring problem in the manuscript:

- Recommend changing first sentence of Abstract to read 'We conducted a clinic-based cross-sectional survey among 710 PLWHA in stable 'sexual' relationships in central and southwestern Uganda.
- Recommend changing 'HIV related' to 'HIV-related' in the Abstract, Methods section and throughout the manuscript.
- Please change 'HIV infected' to 'HIV-infected' throughout the manuscript.
- Please change 'HIV positive' to 'HIV-positive' throughout the manuscript.

Abstract, Methods section: Suggest changing to '…psychosocial factors (maladaptive coping styles, negative life events, social support, resilience, HIV stigma)…'

Inappropriate capitalisation if a recurring problem in the manuscript:

- Recommend changing sentence in Abstract, Methods section to '…and clinical factors (CD4 counts, body weight, height, HIV clinical stage, treatment adherence).'
- Recommend changing to 'negative life events', 'social support', and 'resilience' in Methods, Study variables, fourth sentence.
- Introduction, third paragraph, fifth sentence: Categories of sexual dysfunction do not need to be capitalised.
- Methods, Study variables, fifth sentence: Recommend changing to 'body weight' and 'height'.
- Table 1: 'Others' in 'Moslems & Others' don't need to be capitalised.
- Table 2a, Orgasm problems, second item: 'How' does not need to be capitalised.
- Results, Factors associated with sexual dysfunction, first paragraph, third sentence: 'Depressive' in 'Major Depressive disorder' does not need to be capitalised.

Abstract, first sentence of Results: Suggest changing to 'SD was more prevalent in women (38.7%) than men (17.6%) and the majority (89.3% of men and 66.3% of women)…'

Abstract, first sentence of Conclusion: Suggest changing to 'Sexual dysfunction has considerable prevalence among PLWHA in Uganda. It is associated with socio-demographic, psychiatric and clinical illness factors. To further improve the quality of life of PLWHA, they should be screened for sexual dysfunction as part of routine assessment.'

Introduction, first paragraph, second sentence: Suggest changing to 'As a result, attention has now shifted to…'

Introduction, first paragraph, fourth sentence: Suggest changing to 'Sexual function in the context of HIV/AIDS has mainly been studied with respect to…'

Introduction, first paragraph, fifth sentence: Suggest changing to 'From a public health perspective, sexual problems may predispose individuals to unsafe sexual practices as well as intimate partner relationship discord, including marital violence, and poor ART adherence.

Introduction, first paragraph, seventh sentence: Recommend changing 'ill mental health' to 'poor mental health'.

Introduction, second paragraph, second sentence: Suggest changing to 'It involves the feelings of desire, behavior that brings pleasure to oneself and one's partner, and stimulation of the primary sex organs, including coitus. The activity should be devoid of…'

Introduction, second paragraph, third sentence: Suggest changing to 'Anatomy, physiology, psychology, the culture in which one lives, relationships with others, and the developmental experiences throughout the life cycle determines sexuality.'

Introduction, third paragraph, third sentence: Please replace 'FSD' with 'SD'.

Methods, Study setting, second paragraph, third sentence: Suggest changing to 'On the other hand, TASO Entebbe commenced its work in 1991 and…'

Methods, Study setting, second paragraph, fourth sentence: Suggest changing to 'Entebbe, being urbanized, has an ever-growing and transient population including traders, fish-mongers, and uniformed personnel. It is also a first and last stop for international tourists.'

Methods, Study variables, first paragraph, first sentence: Suggest changing to 'A range of explanatory variables that included socio-demographic, psychiatric illness, and clinical factors were assessed.'

Methods, Study variables, first paragraph, second sentence: Suggest changing '…constructed from common household items…' to '…constructed from having common household items…'

Methods, Study variables, first paragraph, third sentence: Suggest changing to 'The psychiatric illness factors included major depressive disorder and suicidality (assessed using the Mini International Neuropsychiatric interview (M.I.N.I. Plus) (Sheehan and Lecrubier 2006); severity of depressive symptoms was assessed using the Centre for Epidemiological Studies-Depression questionnaire (CES-D).'

Methods, Study variables, first paragraph, fourth sentence: The abbreviation 'COPE' needs to be defined at first mention.

Methods, Study variables, first paragraph, fifth sentence: The reference here, presumably WHO 2007, needs to be reviewed for style.

Methods, Study variables, fifth paragraph, first sentence: Recommend removing 'International Index of Erectile Function' in favour of abbreviation 'IIEF', as this has been previously defined.

Methods, Study variables, fifth paragraph, first sentence: Recommend removing 'Female Sexual Functioning Index' in favour of abbreviation 'FSFI', as this has been previously defined.

Methods, Study variables, fifth paragraph, first sentence: Recommend changing '…and determination of their internal consistency…' to '…and subsequent determination of their internal consistency…'

Methods, Study variables, fifth paragraph, first sentence: Please change 'Cronbach alpha' to 'Cronbach's alpha'.

Methods, Study variables, fifth paragraph, second sentence: Please change 'undertaken' to 'undertake'.

Methods, Study variables, fifth paragraph, third sentence: Please change 'consistence' to 'consistency'.

Methods, Study variables, fifth paragraph, fifth sentence: Again, please remove the whole name of measures in favour of their abbreviations, IIEF and FSFI, since these have been previously defined.

Methods, Statistical analysis, first paragraph, fourth sentence: Please change to "sexual dysfunction for men" and "sexual dysfunction for women" as these are separate variables.

Methods, Statistical analysis, first paragraph, fifth sentence: Suggest changing to 'To investigate the association of sexual dysfunction with socio-demographic, psychosocial and psychiatric illness factors…'

Methods, Statistical analysis, second paragraph: Suggest changing 'at bivariate analyses' to 'with bivariate analyses'.

Results, Participant characteristics, second sentence: Suggest changing to 'The majority of the participants…'

Table 1: 'Moslems' should be 'Muslims'

Table 1: 'ses' should be 'SES' (three instances)

Results, Nature and prevalence of sexual dysfunction, second paragraph, second sentence: Suggest changing to 'The majority of both men (89.3%) and women (66.3%) did not seek help for sexual dysfunction, but more women (25%) sought help from…'

Results, Nature and prevalence of sexual dysfunction, second paragraph, third sentence: Suggest changing 'Of note 3.4% and 6.6%' to 'Of note, 3.4% and 6.6%'.

Table 2a: Please check the bolding of certain words and letters. This does not seem to be for emphasis or, if it is, has been applied incorrectly.

Table 2a, Sexual Pain problems, third item: 'Over the past 4 weeks' is unnecessary as it's indicated at the top of the table.

Table 2b: Please check the underline of certain words. Is this necessary?

Table 2b, Sexual dysfunction, second row: Please delete empty row.

Results, Factors associated with sexual dysfunction, first paragraph, second sentence: Suggest changing to 'A marital status indicated as widowed or separated…'

Results, Factors associated with sexual dysfunction, first paragraph, second sentence: Please change 'Table 3' to 'Table 3 and 4' since these results are the same using bivariate or multivariate analysis.

Results, Factors associated with sexual dysfunction, first paragraph, fourth sentence: Please change 'Table 5' to 'Table 4'. There is no Table 5.

Discussion, general: Please replace 'sexual dysfunction' with 'SD' as the abbreviation is well-defined at this stage.

Discussion, first paragraph, first sentence: Suggest changing to 'We sought to explore the topic of sexual functioning among patients living with HIV in Uganda, a rather taboo topic in this setting, with an aim of informing…'

Discussion, first paragraph, second and third sentences: Suggest changing to 'SD is a relatively common problem experienced by men and women living with HIV in Uganda, with one in five men and two in five women affected. Studies on SD in PLWHA…'

Discussion, first paragraph, fourth sentence: Suggest changing to 'It is worth noting that for both men and women with SD, the majority did not seek treatment. This was especially so for men, with less…'

Discussion, Nature and prevalence of dysfunction among PLWHA, second sentence: Please change 'dysfunctions' to 'dysfunction'.

Discussion, Nature and prevalence of dysfunction among PLWHA, fourth sentence: Suggest changing to 'This finding is similar to what has been reported in previous studies which found that the healthcare system may not be prepared for immediate or continuing support…'

Discussion, Factors associated with sexual dysfunction in HIV infected men, second paragraph, eighth sentence: Please change '2' to 'two'.

Discussion, Factors associated with sexual dysfunction in HIV infected men, second paragraph, ninth sentence: Please remove duplication of 'on the' in 'on the on the other hand'.

Discussion, Factors associated with sexual dysfunction in HIV infected women, second paragraph, second sentence: Suggest changing to 'In our study, a number of sexual problems, relating to reduced desire for and difficulties during sexual activity, were experienced by women. These findings are similar to findings…'

Discussion, Factors associated with sexual dysfunction in HIV infected women, second paragraph, fourth sentence: Suggest changing to 'Concerns about HIV transmission, HIV treatment including ART and its side-effects…'

Discussion, Factors associated with sexual dysfunction in HIV infected women, second paragraph, fifth sentence: Suggest changing to 'This could be a possible explanation why one in three women who participated…'

Discussion, Factors associated with sexual dysfunction in HIV infected women, third paragraph, first sentence: Suggest changing to 'We found that older age (>45 years) and being widowed or separated…'

Discussion, Factors associated with sexual dysfunction in HIV infected women, third paragraph, second sentence: Suggest changing to 'Oyedokun and others (2014) found no association between FSD and religion among HIV-positive women in Nigeria, however, increasing age (Oyedokun, Odeigah et al. 2014), was significantly associated with FSD (Luzi, Guaraldi et al. 2009). These findings are corroborated in our study.'

Discussion, Factors associated with sexual dysfunction in HIV infected women, third paragraph, third sentence: Please add a space after the bracket in '(Luzi, Guaraldi et al. 2009) and one can'.

Discussion, Factors associated with sexual dysfunction in HIV infected women, fifth paragraph, first sentence: Please add a space before the bracket in 'higher (Wilson, Jean-Louis et al. 2010)'.

Discussion, Factors associated with sexual dysfunction in HIV infected women, sixth paragraph, first sentence: Suggest changing to 'Psychosocial variables such as mental health status and quality of life, but not clinical status (illness stage), have been reported…'

References: References should be numbered and their style should be standardised throughout the manuscript.

Abstract

Sentence starting with 'However, it is rarely discussed…': Recommend replacing 'secretive' with 'private'. This seems to be a more appropriate word as sexual life is considered private by many, but not necessarily a secret.

Introduction

Introduction, second paragraph, first sentence: Recommend changing to 'Normal sexuality if a psycho-biological activity necessary for psychological and biological balance in most humans, and for the continuation of human life.' It is dangerous to make blanket statements about sexuality being

necessary for all people as it marginalises those who cannot, or choose not to, engage with their sexuality.

Introduction, third paragraph, section from 'PLWHA are living longer and healthier lives…' up to '…unintended pregnancy (Harrison and O'Sullivan 2010, Okigbo and Speizer 2015, Wasswa, Kabagenyi et al. 2020). In the first instance, the references should be standardised to a single format. In the second instance, this seems to be a repetition and expansion of what was said in the first paragraph of the introduction. Recommend working in the contents of the first paragraph into this one, resulting in the paragraph starting with 'Normal sexuality…' being the first of the Introduction.

Methods

Methods, Study design, second sentence: Please explain why sexual is written in inverted commas ('sexual' relationships). I have not been able to find a rationale for this in the manuscript. It seems unnecessary and could alienate readers.

Methods, Statistical analysis, general: I can appreciate that this may have been difficult to ascertain and potentially impossible to obtain reliable reporting on, given the setting, but did the authors consider subgroup effects by homo- and heterosexual respondents? While I understand potential legal ramifications of exploring this, it should at least be noted in the limitations as important subgroup effects might exist – particularly since major depressive disorder is a risk factor for sexual dysfunction. It is likely that MDD disproportionately affects homosexual people, particularly in a context where they are not free to express their sexuality.

Results

Results, Participant characteristics: Please make some mention of the disproportionate representation of women in the sample. Conclusions based on a sample of 159 men, particularly when juxtaposed or compared with women, could be spurious, and should be noted.

Results, Nature and prevalence of sexual dysfunction, first paragraph, third sentence: 'number of times sexual intercourse was attempted' is listed as a prevalent problem. What does this mean? Is a high number of attempts bad or indicative or sexual dysfunction?

Table 3: It is not clear from this table or the accompanying methodological text why certain parameters are set at 1 with no 95% confidence interval indicated. Were these variables set as such in the model? This needs to be explained.

Table 3: It is not clear why no results of HIV neurocognitive impairment is given for men. This needs to be described, either by a footnote to the table or in text.

Table 4: Please provide the results of stroke from the bivariate model in Table 3, as it is not listed there. Stroke, presumably previous stroke, shows significant association with SD for both men and women, and the results from the bivariate analysis should be shown. In addition, the outcome should be capitalised to 'Stroke' and the significance level for men should be shown as 'P=0.023'.

Table 4: Bivariate analysis of diabetes in men meets the *a priori* cut-off of P=0.2 for inclusion in the multivariate model. Why are these results not presented?

Results, Factors associated with sexual dysfunction: It is not clear why some results from the bivariate model and some results from the multivariate model are reported. Results from the multivariate model, representing the most adjusted measures, should be used preferentially – unless there is a reason not to do so. If the latter is the case, this needs to be explicitly explained. If both results need to be reported, they should be clearly identified according to the model it is derived from.

Results, Factors associated with sexual dysfunction, first paragraph: In addition to other socio-demographic variables listed, employment is also significantly associated with SD in women at the 5% level.

Results, Factors associated with sexual dysfunction, first paragraph: It is not clear why only some numeric results from statistical models are reported in text.

Results, Factors associated with sexual dysfunction, first paragraph, third sentence: Please change '0.61' to '1.61' in accordance with the result in Table 4.

Results, Factors associated with sexual dysfunction, second paragraph, first sentence: Suggest changing to 'With respect to men, religion (being of the Muslim or another faith) was the only…'

Results, Factors associated with sexual dysfunction, second paragraph: In addition to other socio-demographic variables listed, age is also highly significantly associated with SD in men. Why is this not mentioned here?

Results, Factors associated with sexual dysfunction, second paragraph: Stroke is also significantly associated with SD in men according to the results presented in Table 4. This needs to be reported here, particularly in the light of the role of the vascular system in erectile function.

Results, Factors associated with sexual dysfunction, third paragraph: It is not clear why this information is presented here. The text relating to the ages of respondents should be placed at the start of the Results section, though the age of male respondents should also be reported. Furthermore, the categorisation of education and religion should be described in the Methods section. Finally, it is not clear why this section describes the categorisation of religion as 'Christian and non-Christian' when all other sections report 'Christian' and 'Muslim and others'.

Discussion

Discussion, Factors associated with sexual dysfunction in HIV infected men, general: The authors state that their study did not find associations with physical or anthropometric factors and SD. However, both stroke (in the multivariate model) and diabetes (in the bivariate model, with no results for multivariate analysis despite meeting the *a priori* cut-off) are significantly associated with male SD. Both of these also have known vascular causes and/or effects; the vascular system, in turn, is arguably the most important system for erectile function. As such, the statement that no associations between physical factors and SD were found is patently incorrect.

Discussion, Factors associated with sexual dysfunction in HIV infected men, second paragraph, sixth sentence: It is not clear what the (10) at the end of this sentence refers to. The next sentence seems to follow on from this, stating 'In that study', but reference 10 in the manuscript does not correspond to Lallemand, Salhi et al. 2002.

Discussion, Factors associated with sexual dysfunction in HIV infected men, second paragraph, eighth sentence: The authors state the no association was found with ART, however, 'ART' as an explanatory variable was not measured – only ART adherence. Please specify here whether ART adherence is the intention, or whether some other dimension of 'ART' was measured and its association tested.

Discussion, Factors associated with sexual dysfunction in HIV infected women, third paragraph, general: It is noticeable how much discussion is had around the causal pathways of age in SD in women living with HIV, yet nothing is said about the effect of age for men. This is a glaring omission.

Limitations

General: As noted previously, it is acknowledged that the legal environment in Uganda may have precluded the accurate gathering of information around homo- and heterosexuality among the sample. However, there may be important subgroup effects and this possibility should be noted here.

Author contributions

General: The authors state that detailed contributions are to be listed under the section in the second sentence, however, no such information is forthcoming. The only explicit contribution that is listed is analysis and interpretation by WS. This is not sufficient detail to determine whether ICMJE authorship criteria have been met.
