# Supplementary material for: A ‘hidden problem’: Nature, prevalence and factors associated with sexual dysfunction in persons living with HIV/AIDS in Uganda
Source: PLoS One. 2024 Mar 7;19(3):e0295224. doi: 10.1371/journal.pone.0295224 (PMC10919729; doi:10.1371/journal.pone.0295224)
Supplement: Attachment — Submitted filename: Response to Review comments.pdf [file pone.0295224.s002.pdf]

Dear Sir/Madam,

Response to review comments

**Comment**	**Response**
General<br>Abstract, sentence starting with 'Among individuals living with HIV/AIDS…': Suggest adding changing<br>to '…have hardly been studied, especially in sub-Saharan Africa…'	Thank you for the comment. The change has been effected as advised. Page 1
[Lack of] hyphenation is a recurring problem in the manuscript:<br>Recommend changing first sentence of Abstract to read 'We conducted a clinic-based cross-sectional<br>survey among 710 PLWHA in stable 'sexual' relationships in central and southwestern<br>Uganda.	The first sentence of abstract has been changed to read as: We conducted a clinic-based cross-sectional survey among 710 people living with HIV/AIDS in stable 'sexual' relationships in central and southwestern Uganda.
Recommend changing 'HIV related' to 'HIV-related' in the Abstract, Methods section and<br>throughout the manuscript	'HIV related' has been changed to 'HIV-related' throughout the manuscript
Please change 'HIV infected' to 'HIV-infected' throughout the manuscript.	'HIV infected' has been changed to 'HIV-infected' throughout the manuscript
Please change 'HIV positive' to 'HIV-positive' throughout the manuscript.	'HIV positive' has been changed to 'HIV-positive' throughout the manuscript
Abstract, Methods section: Suggest changing to '…psychosocial factors (maladaptive coping styles,<br>negative life events, social support, resilience, HIV stigma)…'	The change has been effected as advised Page 1
Inappropriate capitalisation is a recurring problem in the manuscript: Recommend changing sentence in Abstract, Methods section to '…and clinical factors (CD4 counts, body weight, height, HIV clinical stage, treatment adherence).'	The sentence has been changed as recommended
Recommend changing to 'negative life events', 'social support', and 'resilience' in Methods,<br>Study variables, fourth sentence.	The sentence has been changed as recommended
Introduction, third paragraph, fifth sentence: Categories of sexual dysfunction do not need to be capitalised.	Thank you for the observation. The capitalisation has been removed
Methods, Study variables, fifth sentence: Recommend changing to 'body weight' and 'height'.	Thank you. The change has been effected as recommended
Table 1: 'Others' in 'Moslems & Others' don't need to be capitalised.	The capitalisation has been removed
Table 2a, Orgasm problems, second item: 'How' does not need to be capitalised.	The capitalisation has been removed

Results, Factors associated with sexual dysfunction, first paragraph, third sentence: ‘Depressive’ in ‘Major Depressive disorder’ does not need to be capitalised.	The capitalisation has been removed
Abstract, first sentence of Results: Suggest changing to ‘SD was more prevalent in women (38.7%) than men (17.6%) and the majority (89.3% of men and 66.3% of women)…’	The change has been effected as suggested
Abstract, first sentence of Conclusion: Suggest changing to ‘Sexual dysfunction has considerable prevalence among PLWHA in Uganda. It is associated with socio-demographic, psychiatric and clinical illness factors. To further improve the quality of life of PLWHA, they should be screened for sexual dysfunction as part of routine assessment.’	The change has been effected as recommended
Introduction, first paragraph, second sentence: Suggest changing to ‘As a result, attention has now shifted to…’	The change has been effected as recommended
Introduction, first paragraph, fourth sentence: Suggest changing to ‘Sexual function in the context of HIV/AIDS has mainly been studied with respect to…’	The change has been effected as recommended
Introduction, first paragraph, fifth sentence: Suggest changing to ‘From a public health perspective, sexual problems may predispose individuals to unsafe sexual practices as well as intimate partner relationship discord, including marital violence, and poor ART adherence.	The change has been effected as recommended
Introduction, first paragraph, seventh sentence: Recommend changing ‘ill mental health’ to ‘poor mental health’.	The change has been effected as recommended
Introduction, second paragraph, second sentence: Suggest changing to ‘It involves the feelings of desire, behavior that brings pleasure to oneself and one’s partner, and stimulation of the primary sex organs, including coitus. The activity should be devoid of…’	The change has been effected as recommended
Introduction, second paragraph, third sentence: Suggest changing to ‘Anatomy, physiology, psychology, the culture in which one lives, relationships with others, and the developmental experiences throughout the life cycle determines sexuality.’<br>Introduction, third paragraph, third sentence: Please replace ‘FSD’ with ‘SD’.	The change has been effected as recommended
Methods, Study setting, second paragraph, third sentence: Suggest changing to ‘On the other hand, TASO Entebbe commenced its work in 1991 and…’	The change has been effected as recommended
Methods, Study setting, second paragraph, fourth sentence: Suggest changing to ‘Entebbe, being urbanized, has an ever-growing and transient population including traders, fish-mongers, and uniformed personnel. It is also a first and last stop for international tourists.’	The change has been effected as recommended
Methods, Study variables, first paragraph, first sentence: Suggest changing to ‘A range of explanatory variables that included socio-demographic, psychiatric illness, and clinical factors were assessed.’	The change has been effected as recommended
Methods, Study variables, first paragraph, second sentence: Suggest changing ‘…constructed from common household items…’ to ‘…constructed from having common household items…’	The change has been effected as recommended

Methods, Study variables, first paragraph, third sentence: Suggest changing to 'The psychiatric illness factors included major depressive disorder and suicidality (assessed using the Mini International Neuropsychiatric interview (M.I.N.I. Plus) (Sheehan and Lecrubier 2006); severity of depressive symptoms was assessed using the Centre for Epidemiological Studies-Depression questionnaire (CESD).'	The change has been effected as recommended
Methods, Study variables, first paragraph, fourth sentence: The abbreviation 'COPE' needs to be defined at first mention.	The abbreviation COPE has been defined as 'Coping Orientation to Problems Experienced'
Methods, Study variables, first paragraph, fifth sentence: The reference here, presumably WHO 2007, needs to be reviewed for style.	The reference has been revised to WHO 2007
Methods, Study variables, fifth paragraph, first sentence: Recommend removing 'International Index of Erectile Function' in favour of abbreviation 'IIEF', as this has been previously defined.	The change has been effected as recommended
Methods, Study variables, fifth paragraph, first sentence: Recommend removing 'Female Sexual Functioning Index' in favour of abbreviation 'FSFI', as this has been previously defined.	The change has been effected as recommended
Methods, Study variables, fifth paragraph, first sentence: Recommend changing '…and determination of their internal consistency…' to '…and subsequent determination of their internal consistency…'	The change has been effected as recommended
Methods, Study variables, fifth paragraph, first sentence: Please change 'Cronbach alpha' to 'Cronbach's alpha'.	The change has been effected as recommended
Methods, Study variables, fifth paragraph, second sentence: Please change 'undertaken' to 'undertake'.	The change has been effected as recommended
Methods, Study variables, fifth paragraph, third sentence: Please change 'consistence' to 'consistency'.	The change has been effected as recommended
Methods, Study variables, fifth paragraph, fifth sentence: Again, please remove the whole name of measures in favour of their abbreviations, IIEF and FSFI, since these have been previously defined.	The change has been effected as recommended
Methods, Statistical analysis, first paragraph, fourth sentence: Please change to "sexual dysfunction for men" and "sexual dysfunction for women" as these are separate variables.	The change has been effected as recommended
Methods, Statistical analysis, first paragraph, fifth sentence: Suggest changing to 'To investigate the association of sexual dysfunction with socio-demographic, psychosocial and psychiatric illness factors…'	The change has been effected as recommended
Methods, Statistical analysis, second paragraph: Suggest changing 'at bivariate analyses' to 'with bivariate analyses'.	The change has been effected as recommended
Results, Participant characteristics, second sentence: Suggest changing to 'The majority of the participants…'	The change has been effected as recommended
Table 1: 'Moslems' should be 'Muslims'	The change has been effected as recommended
Table 1: 'ses' should be 'SES' (three instances)	The change has been effected as recommended
Results, Nature and prevalence of sexual dysfunction, second paragraph, second sentence: Suggest changing to 'The majority of both men (89.3%) and women (66.3%) did not seek help for sexual dysfunction, but more women (25%) sought help from…'	The change has been effected as recommended
Results, Nature and prevalence of sexual dysfunction, second paragraph, third sentence: Suggest changing 'Of note 3.4% and 6.6%' to 'Of note, 3.4% and 6.6%'.	The change has been effected as recommended

Table 2a: Please check the bolding of certain words and letters. This does not seem to be for emphasis or, if it is, has been applied incorrectly.	The bolding has been removed
Table 2a, Sexual Pain problems, third item: 'Over the past 4 weeks' is unnecessary as it's indicated at the top of the table.	It has been removed
Table 2b: Please check the underline of certain words. Is this necessary?	The underline has been removed
Table 2b, Sexual dysfunction, second row: Please delete empty row.	The empty row has been deleted
Results, Factors associated with sexual dysfunction, first paragraph, second sentence: Suggest changing to 'A marital status indicated as widowed or separated…'	The change has been effected as recommended
Results, Factors associated with sexual dysfunction, first paragraph, second sentence: Please changes 'Table 3' to 'Table 3 and 4' since these results are the same using bivariate or multivariate analysis.	'Table 3' has been changed to 'Table 3 and 4'
Results, Factors associated with sexual dysfunction, first paragraph, fourth sentence: Please change 'Table 5' to 'Table 4'. There is no Table 5.	'Table 5' has been changed to 'Table 4'
Discussion, general: Please replace 'sexual dysfunction' with 'SD' as the abbreviation is well-defined at this stage.	The change has been effected as recommended
Discussion, first paragraph, first sentence: Suggest changing to 'We sought to explore the topic of sexual functioning among patients living with HIV in Uganda, a rather taboo topic in this setting, with an aim of informing…'	The change has been effected as recommended
Discussion, first paragraph, second and third sentences: Suggest changing to 'SD is a relatively common problem experienced by men and women living with HIV in Uganda, with one in five men and two in five women affected. Studies on SD in PLWHA…'	The change has been effected as recommended
Discussion, first paragraph, fourth sentence: Suggest changing to 'It is worth noting that for both men and women with SD, the majority did not seek treatment. This was especially so for men, with less…'	The change has been effected as recommended
Discussion, Nature and prevalence of dysfunction among PLWHA, second sentence: Please change 'dysfunctions' to 'dysfunction'.	The change has been effected as recommended
Discussion, Nature and prevalence of dysfunction among PLWHA, fourth sentence: Suggest changing to 'This finding is similar to what has been reported in previous studies which found that the healthcare system may not be prepared for immediate or continuing support…'	The change has been effected as recommended
Discussion, Factors associated with sexual dysfunction in HIV infected men, second paragraph, and eighth sentence: Please change '2' to 'two'.	The change has been effected as recommended
Discussion, Factors associated with sexual dysfunction in HIV infected men, second paragraph, ninth Sentence: Please remove duplication of 'on the' in 'on the on the other hand'.	The change has been effected as recommended
Discussion, Factors associated with sexual dysfunction in HIV infected women, second paragraph, second sentence: Suggest changing to 'In our study, a number of sexual problems, relating to reduced desire for and difficulties during sexual activity, were experienced by women. These findings are similar to findings…'	The change has been effected as recommended
Discussion, Factors associated with sexual dysfunction in HIV infected women, second paragraph, fourth sentence: Suggest changing to 'Concerns about HIV transmission, HIV treatment including ART and its	The change has been effected as recommended

side-effects…’	
Discussion, Factors associated with sexual dysfunction in HIV infected women, second paragraph, and fifth sentence: Suggest changing to ‘This could be a possible explanation why one in three women who participated…’	The change has been effected as recommended
Discussion, Factors associated with sexual dysfunction in HIV infected women, third paragraph, and first sentence: Suggest changing to ‘We found that older age (>45 years) and being widowed or separated…’	The change has been effected as recommended
Discussion, Factors associated with sexual dysfunction in HIV infected women, third paragraph, second sentence: Suggest changing to ‘Oyedokun and others (2014) found no association between FSD and religion among HIV-positive women in Nigeria, however, increasing age (Oyedokun, Odeigah et al. 2014), was significantly associated with FSD (Luzi, Guaraldi et al. 2009). These findings are corroborated in our study.’	The change has been effected as recommended
Discussion, Factors associated with sexual dysfunction in HIV infected women, third paragraph, third sentence: Please add a space after the bracket in ‘(Luzi, Guaraldi et al. 2009) and one can’.	A space has been added
Discussion, Factors associated with sexual dysfunction in HIV infected women, fifth paragraph, first sentence: Please add a space before the bracket in ‘higher (Wilson, Jean-Louis et al. 2010)’.	The change has been effected as recommended
Discussion, Factors associated with sexual dysfunction in HIV infected women, sixth paragraph, first sentence: Suggest changing to ‘Psychosocial variables such as mental health status and quality of life, but not clinical status (illness stage), have been reported…’	The change has been effected as recommended
References: References should be numbered and their style should be standardised throughout the manuscript.	The references have been numbered
Abstract	
Sentence starting with ‘However, it is rarely discussed…’: Recommend replacing ‘secretive’ with ‘private’. This seems to be a more appropriate word as sexual life is considered private by many, but not necessarily a secret.	The change has been effected as recommended
Introduction	
Introduction, second paragraph, first sentence: Recommend changing to ‘Normal sexuality if a psychobiological activity necessary for psychological and biological balance in most humans, and for the continuation of human life.’ It is dangerous to make blanket statements about sexuality being necessary for all people as it marginalises those who cannot, or choose not to, engage with their sexuality.	The change has been effected as recommended
Introduction, third paragraph, section from ‘PLWHA are living longer and healthier lives…’ up to‘…unintended pregnancy (Harrison and O’Sullivan 2010, Okigbo and Speizer 2015, Wasswa, Kabagenyi et al. 2020). In the first instance, the references should be standardised to a single format. In the second instance, this seems to be a repetition and expansion of what was said in the first paragraph of the introduction. Recommend working in the contents of the first paragraph into this one, resulting in the paragraph starting with ‘Normal sexuality…’ being the first of the Introduction.	The section has been revised and the sentence on normal sexuality is now the first one in the introduction
Methods	
Methods, Study design, the second sentence: Please explain why sexual	Thank you for the comment.

is written in inverted commas ('sexual' relationships). I have not been able to find a rationale for this in the manuscript. It seems unnecessary and could alienate readers.	The inverted commas have been removed.
Methods, Statistical analysis, general: I can appreciate that this may have been difficult to ascertain and potentially impossible to obtain reliable reporting on, given the setting, but did the authors consider subgroup effects by homo- and heterosexual respondents? While I understand potential legal ramifications of exploring this, it should at least be noted in the limitations as important subgroup effects might exist – particularly since major depressive disorder is a risk factor for sexual dysfunction. It is likely that MDD disproportionately affects homosexual people, particularly in a context where they are not free to express their sexuality.	We were not able to conduct subgroup analyses by homo- and heterosexual respondents. In Uganda, homosexuality is still a sensitive issue to discuss.
Results	
Results, Participant characteristics: Please make some mention of the disproportionate representation of women in the sample. Conclusions based on a sample of 159 men, particularly when juxtaposed or compared with women, could be spurious, and should be noted.	A sentence has been added to the 3rd paragraph in the discussion section 'However, the number of men was smaller than that of women in this study. This could have affected the results.'
Results, Nature and prevalence of sexual dysfunction, first paragraph, third sentence: 'number of times sexual intercourse was attempted' is listed as a prevalent problem. What does this mean? Is a high number of attempts bad or indicative or sexual dysfunction?	The number of times sexual intercourse was attempted was measured as" no attempts, one to two attempts/ three to four attempts, five to six attempts, seven to ten attempts/ Eleven or more attempts". In this case, fewer sexual attempts were assumed to indicate lower sex drive and, therefore sexual dysfunction.
Table 3: It is not clear from this table or the accompanying methodological text why certain parameters are set at 1 with no 95% confidence interval indicated. Were these variables set as such in the model? This needs to be explained.	The parameters that were set at 1 are the comparison factors. The odds of these other parameters/categories are interpreted in relation to the comparison parameters
Table 3: It is not clear why no results of HIV neurocognitive impairment are given for men. This needs to be described, either by a footnote to the table or in text.	* HIV neurocognitive impairment among men was not included due to perfect collinearity. This has been added as a footnote
Table 4: Please provide the results of stroke from the bivariate model in Table 3, as it is not listed there. Stroke, presumably previous stroke, shows significant association with SD for both men and women, and the results from the bivariate analysis should be shown. In addition, the outcome should be capitalised to 'Stroke' and the significance level for men should be shown as 'P=0.023'.	Thank you for the observation. The results have been included in table 3
Table 4: Bivariate analysis of diabetes in men meets the a priori cut-off	Some of the factors met the

of P=0.2 for inclusion in the multivariate model. Why are these results not presented?	apriori cut-off. However they presented multicollinearity in the multivariate model and therefore reducing precision of the estimates and were therefore eliminated.
Results, Factors associated with sexual dysfunction: It is not clear why some results from the bivariate model and some results from the multivariate model are reported. Results from the multivariate model, representing the most adjusted measures, should be used preferentially – unless there is a reason not to do so. If the latter is the case, this needs to be explicitly explained. If both results need to be reported, they should be clearly identified according to the model it is derived from.	Some of the factors met the apriori cut-off. However they presented multicollinearity in the multivariate model and therefore reducing precision of the estimates and were therefore eliminated.
Results, Factors associated with sexual dysfunction, first paragraph: In addition to other sociodemographic variables listed, employment is also significantly associated with SD in women at the 5% level.	This has been indicated
Results, Factors associated with sexual dysfunction, first paragraph: It is not clear why only some numeric results from statistical models are reported in text.	The details of the results are in the table. It would have been repetition to report all results in text and table
Results, Factors associated with sexual dysfunction, first paragraph, third sentence: Please change '0.61' to '1.61' in accordance with the result in Table 4.	Thank you for the observation. This has been addressed
Results, Factors associated with sexual dysfunction, second paragraph, first sentence: Suggest changing to 'With respect to men, religion (being of the Muslim or another faith) was the only…'	This has been changed. Thanks for the comment. This has been addressed
Results, Factors associated with sexual dysfunction, second paragraph: In addition to other sociodemographic variables listed, age is also highly significantly associated with SD in men. Why is this not mentioned here?	Age has been included. Thank you for the observation
Results, Factors associated with sexual dysfunction, second paragraph: Stroke is also significantly associated with SD in men according to the results presented in Table 4. This needs to be reported here, particularly in the light of the role of the vascular system in erectile function.	It has been included. Thank you
Results, Factors associated with sexual dysfunction, third paragraph: It is not clear why this information is presented here. The text relating to the ages of respondents should be placed at the start of the Results section, though the age of male respondents should also be reported	The information has been removed from this section and taken to methods and the beginning of the results section
Furthermore, the categorisation of education and religion should be described in the Methods section.	This has been taken to the end of the methods section
Finally, it is not clear why this section describes the categorisation of religion as 'Christian and non-Christian' when all other sections report 'Christian' and 'Muslim and others'.	This has been harmonised. Religion is categorised as Christian and, muslim and other religion
Discussion	
Discussion, Factors associated with sexual dysfunction in HIV infected men, general: The authors state that their study did not find	Thank you so much for the observarion . it was a wrong

associations with physical or anthropometric factors and SD. However, both stroke (in the multivariate model) and diabetes (in the bivariate model, with no results for multivariate analysis despite meeting the a priori cut-off) are significantly associated with male SD. Both of these also have known vascular causes and/or effects; the vascular system, in turn, is arguably the most important system for erectile function. As such, the statement that no associations between physical factors and SD were found is patently incorrect.	statement and it has been corrected. Indeed, diabetes and stroke and known risk factors for sexual dysfunction. In our results, they were associated with SD.
Discussion, Factors associated with sexual dysfunction in HIV infected men, second paragraph, sixth sentence: It is not clear what the (10) at the end of this sentence refers to. The next sentence seems to follow on from this, stating 'In that study', but reference 10 in the manuscript does not correspond to Lallemand, Salhi et al. 2002.	The mistake has been corrected. The reference is Lallemand et al 2002
Discussion, Factors associated with sexual dysfunction in HIV infected men, second paragraph, eighth sentence: The authors state the no association was found with ART, however, 'ART' as an explanatory variable was not measured – only ART adherence. Please specify here whether ART adherence is the intention, or whether some other dimension of 'ART' was measured and its association tested.	Thank you for the comment. The section refers to ART adherence and has been edited
Discussion, Factors associated with sexual dysfunction in HIV infected women, third paragraph, general: It is noticeable how much discussion is had around the causal pathways of age in SD in women living with HIV, yet nothing is said about the effect of age for men. This is a glaring omission.	Thank you for this comment. A section has been added on the effect of age on sexual dysfunction among men.
Limitations	
General: As noted previously, it is acknowledged that the legal environment in Uganda may have precluded the accurate gathering of information around homo- and heterosexuality among the sample. However, there may be important subgroup effects and this possibility should be noted here.	Thank you for the comment. We did not do subgroup analysis. It is possible that there could have been important subgroup effects
Author contributions	
General: The authors state that detailed contributions are to be listed under the section in the second sentence, however, no such information is forthcoming. The only explicit contribution that is listed is analysis and interpretation by WS. This is not sufficient detail to determine whether ICMJE authorship criteria have been met.	Thank you for this reminder. It has been updated
