# Supplementary material for: A ‘hidden problem’: Nature, prevalence and factors associated with sexual dysfunction in persons living with HIV/AIDS in Uganda
Source: PLoS One. 2024 Mar 7;19(3):e0295224. doi: 10.1371/journal.pone.0295224 (PMC10919729; doi:10.1371/journal.pone.0295224)
Supplement: Attachment — Submitted filename: Response to review comments Oct 23.pdf [file pone.0295224.s003.pdf]

October 27, 2023

Dr. Habil Otanga, Ph.D
Academic Editor
PLOS ONE

Dear Dr. Otanga,

Re: PONE-D-22-24725R1: A ‘hidden problem’: Nature, prevalence and factors associated with sexual dysfunction in persons living with HIV/AIDS in Uganda

Thank you for the opportunity to resubmit this manuscript. Your effort and that of the reviewers is highly appreciated. Here below is a point-by-point response to the review comments.

Comment	Response	Page with changes
**Editor’s comments**<br><br>1. Your work suffers serious editorial shortcomings, from grammar to in-text citations to referencing style.	Thak you for your comment. The manuscript has been revised and improved.	Throughout the manuscript
2. Methods: ED was measured using 6 items on the IIEF. How was the cut-off score of 25 derived (on a 5-point Likert Scale) which is erroneously presented as a 6-point Likert Scale (Section on Study Variables Line 27)? What informed the cut-off score?<br>Reviewers found fault with sampling techniques and determination of sample size, data collection tools and techniques.<br>Why was the definition for SD different for men and women judging by the fact that authors used a single subscale for men (6 items) and the total FSFI score for women?	This has been corrected to a 15 item tool.<br>the cut off score was informed by a previous study (IIEF (Rosen, Riley et al. 1997))<br><br>The definition from ISD is different as assessed differently by the different tools i.e IIEF & FSFI	Page 7

3. Discussion/Results: All tables must be presented in APA format<br>-If scales were used, one would expect Table 2 (a, b) to present frequencies/means of variables and not items. Why were the tables presented item by item?	The APA format has been implemented	Pages 11, 12,13,14,15,16,17,18
When do variables cease to be items and become variables for analysis? What was used in analysis - items in the questionnaire or variables?	Items from the questionnaire were used to generate composite variables for the outcomes.<br><br>Standard variables like sociodemographic were also used.	
Reviewers suggest that authors discuss the statistical part of how categorical and continuous data are reported	This has been added	Page 10
Reviewer #2: Thanks for your paper. Whereas the paper presents an amazing perspective to sexual dysfunction among persons living with HIV in Uganda, there are issues that you need to address:<br>1. Editing: Your entire work suffers from poor editorial work. Words that are not proper nouns start with capital letters e.g., Line 2 of abstract.<br>-Line 2 of abstract is either incomplete or poorly punctuated (Sentence beginning with "Although"	Thank you for the observation. This has been addressed in the revised manuscript	
2. Citations: You need to revise the entire work to be in line with the appropriate journal citation format.<br>Introduction: Line 5<br>Paragraph 2 of Introduction: Line 1, 3, 7, 9<br>Paragraph 3: Line 5, 8, 10, 12. for instance what citation style is "Wilson, Jean-Louis et al 2010"? Decide who the first author is,	Thank you for the guidance. The citations have been revised as advised.	

use their surname followed by et al and year of publication.		
3. Methods: ED was measured using 6 items on the IIEF. How was the cut-off score of 25 derived (on a 5 point LS) which is erroneously presented as a 6 point LS (Section on Study Variables Line 27)? What informed the cut-off score?<br>Why was the definition for SD different for men and women judging by the fact that using a single subscale for men (6 items) and the total FSFI score for women?	This has been addressed in comment 2 above.	
4. Results: All tables must be presented in APA format<br>-If scales were used, one would expect Table 2 (a, b) to present frequencies/means of variables and not items. Why were the tables presented item by item? When do variables cease to be items and become variables for analysis? What was used in analysis - items in the questionnaire or variables?	This has been addressed. All tables are in APA format	Pages 11, 12,13,14,15,16,17,18
5. References do not follow any distinct style. Check PLoS ONE requirements for presenting references.	The references have been revised	
Reviewer #3:<br>Abstract:<br>- By taking into account the study period, sampling technique, data collection tools, and statistical methodologies (descriptive or inferential statistics), the authors needed to update the methods part of the abstract once more in light of their objectives.	The methods section of the abstract has been revised. We added that 'Statistical analyses were conducted using Stata release 15, separately for males and females. Frequencies to the FSFI and IIEF were estimated with their percentages. Categorical variables were described using frequencies and percentages while continuous variables were described using median	

	(IQR). Two binary outcome variables “sexual dysfunction for men and sexual dysfunction for women” were generated. To investigate the association of sexual dysfunction with socio-demographic and psychosocial and psychiatric illness factors, a two-step procedure was adopted. During the first step, bivariate associations between each of the outcome variables and the independent variables were assessed using simple logistic regression models. In the second step, those socio-demographic, psychosocial and psychiatric illness factors that attained a level of significance of P ≤ 0.2 (liberal cut-off point) with bivariate analyses were included in the final multivariate logistic regression models that assessed for the factors that were independently significantly associated with sexual dysfunction. We were not able to conduct subgroup analyses by homo- and heterosexual respondents)	
- The authors must indicate the percentage with a 95% confidence interval for the prevalence of sexual disfunction for males and females, respectively.	This has been incorporated	Page 12
- In light of their findings, the authors ought to revise their recommendations.	Although SD exists in both female and male patients with HIV, there need for specific interventions for the different genders.	

Introduction:<br>- The introduction section focuses primarily on the definition, nature, and determinants of sexual dysfunction, with little to no discussion of its prevalence. I should suggest that the authors review the global literature on the prevalence in order to briefly explain the epidemiology (the distribution) of sexual dysfunction.	The section has been revised to include information on the global burden of SD	5
Methods:<br>-When was this study done? considering that the information came from a secondary source and that the patients with HIV/AIDS were chosen from medical records from what years?		
- In addition to mentioning that this research was conducted as part of Prof Eugene Kinyanda's Senior EDCTP Fellowship funded study entitled, 'Clinical trials in HIV/AIDS in Africa: Should they routinely control for mental health factors?', I believe the authors missed an important section of the materials and methods section, such as how the participants for the study were selected (sampling technique) and the determination of sample size, data collection tools and techniques, the operational definitions of some concepts, etc.	Thank you for the observation. More details have been added on page 7 of the revised manuscript	7
- The authors should thoroughly discuss the statistical part of how categorical and continuous data are reported, respectively, and for variables in the final multivariate model, the authors should plan the adjusted odds	This has been done	Page 12

ratio (AOR) along with the 95% confidence interval. When measuring the outcome variable, the authors needed to clearly categorize it into binary data by coding it.		
- The authors had to identify at what level of significance the variables should be deemed significant in the final multivariate model, which also needed to be assessed and presented together with an AOR and a 95% confidence interval.	This has been done on the multi variate model.	Page 18
- Additionally, the authors had to disclose the precise categorization (their levels) of each independent variable measured in addition to stating the scales.<br>Results:	This has been added	Page 11
- I'm not sure why the authors chose to use the median rather than the mean when reporting the age.	We chose the median over mean because the mean did not accurately reflect the central tendency	
- The authors had to specify what low and high socioeconomic status meant when classifying socioeconomic status based on the wealth index in the method section before reporting here.	This has been added	Page 7
- It is required to give the mean, plus or minus standard deviation, or the sub-total frequency of each domain of measurement scales when reporting sexual dysfunction in males and females. Additionally, for each outcome variable, the authors needed to indicate the 95% confidence interval.	This was reported using prevalence and 95% confidence intervals.	Page 12
- I'll advise you to place the subheading factors related to	This has been done	Pages 16 and 17

sexual dysfunction before presenting the tables labelled "bivariate analysis of related factors (not adjusted) and "multivariate model (tables 3 and 4).		
Discussion:<br>- The finding was well discussed by the authors. The authors were also required to expand on their discussion by including earlier prevalence research related to sexual dysfunction as well as further implications of the study for low-income nations like those in sub-Saharan Africa.	This has been done	Page 26 and 27
- Although it appears that the authors are talking about qualitative findings in their discussion, I believe that they should concentrate on quantitative results instead because they evaluated the study in a quantitative manner, unless they felt the need to include qualitative data.<br>Conclusion and recommendation:	The study did not include any qualitative analyses – this was highlighted in the limitations	
- The authors should have provided a more thorough summary of the main findings and recommendations based on those findings, particularly for those stakeholders working on behavioural changes related to HIV/AIDS and for concerned policymakers.<br>At last, the authors produced some excellent work, but they should pay particular attention to the methods section because the majority of its components are missing.	This has been included	Page 26
